# Oligodendroglioma: Advances in Molecular Mechanisms and Immunotherapeutic Strategies

**DOI:** 10.3390/biomedicines13051133

**Published:** 2025-05-07

**Authors:** Yongxin Zhao, Yan Yu, Weizhi Chen, Xiaojun Zhang, Jing Lv, Heping Zhao

**Affiliations:** Department of Clinical Laboratory, Honghui Hospital, Xi’an Jiaotong University, Xi’an 710054, China; yongxin_0452@163.com (Y.Z.); yu.yan74@163.com (Y.Y.); 18009212715@163.com (W.C.); xiaojunzhangtg@163.com (X.Z.)

**Keywords:** oligodendroglioma, tumor microenvironment, immunotherapy, oncolytic virus therapy, CAR T-cell therapy, immune checkpoint blockade therapy, cancer vaccines

## Abstract

Oligodendroglioma is a central nervous system tumor defined by *IDH1/2* mutations and 1p/19q co-deletion. Current management involves maximal resection followed by radiotherapy/chemotherapy, yielding a 20-year survival rate of 37% for grade 3 tumors according to the WHO 2021 classification. As these tumors primarily affect young to middle-aged patients, novel therapies are urgently needed to improve outcomes. Immunotherapy has revolutionized tumor treatment by modulating immune responses. However, its application in oligodendrogliomas faces two major hurdles, including the immunosuppressive tumor microenvironment (TME) and the blood–brain barrier’s restrictive properties. This review first examines oligodendroglioma’s molecular alterations to refine diagnosis and guide targeted therapies. Next, we focus on the oligodendroglioma TME to evaluate emerging immunotherapies, including oncolytic viruses, immune checkpoint blockade, chimeric antigen receptor (CAR) T-cell therapy, and cancer vaccines. Finally, we discuss current challenges and future directions to overcome therapeutic limitations and advance treatment strategies.

## 1. Introduction

Gliomas represent the most prevalent primary solid tumors of central nervous system (CNS) malignancies [1], and are classified into four grades based on distinct histological and molecular characteristics according to the 2021 World Health Organization (WHO) CNS classification [2]. Specifically, the CNS WHO grade 2 low-grade gliomas (LGGs) include oligodendrogliomas and astrocytomas, which predominantly affect young adults during the third to fourth decades of life [3]. LGGs may transform malignantly into more aggressive grade 3 or 4 gliomas, recognized as high-grade gliomas (HGGs) [3,4]. Oligodendrogliomas, known as neuroepithelial tumors originating in the CNS, is a rare subtype of diffuse gliomas, and represent the third most common primary brain tumor, following glioblastoma and diffuse astrocytoma and accounting for approximately 5% of all primary CNS tumors [5,6]. In the United States, the incidence of oligodendrogliomas is estimated at around 1000 new cases per year [7]. According to the 2021 WHO CNS classification guidelines, oligodendrogliomas are molecularly characterized by mutations in the isocitrate dehydrogenase (*IDH*) and the co-deletion of chromosome arms 1p and 19q [2,8]. Currently, the standard management for oligodendrogliomas involves maximal surgical resection to remove as much of the tumor as possible, followed by postoperative radiation [9]. However, the frequent localization of these tumors in the frontal lobe complicates surgical excision and increases the risk of damage to critical brain regions [7]. For recurrent oligodendrogliomas, reoperation and chemotherapy are the primary therapeutic options, although the efficacy remains limited, often resulting in suboptimal outcomes [7].

Similar to other tumors, oligodendrogliomas develop due to molecular alterations, including genetic mutations, some of which hold significant diagnostic and prognostic values, serving as essential biomarkers for tumor detection and classification [6]. LGGs frequently harbor mutations in the *IDH* gene [10]; *IDH*-mutant LGGs can be further divided into two subtypes based on the presence of the 1p/19q co-deletion: oligodendroglioma (with the co-deletion) and astrocytoma (without the co-deletion) [11]. Despite advancements in molecular biology, our understanding of the biological mechanisms and molecular pathways driving oligodendrogliomas remains incomplete.

The tumor microenvironment (TME) comprises a complex network of innate and adaptive immune cells, along with various immune molecules [12]. As tumor immunogenicity evolves constantly and TME becomes increasingly immunosuppressive, tumor cells can evade immune surveillance and proliferate unchecked [12]. Tumor immunotherapy aims to rejuvenate anti-tumor immune cells, counteract immune evasion mechanisms, and harness the immune system to restore anti-tumor immunity. Immunotherapy has demonstrated the potential to elicit robust and durable immune responses against tumors [13]. The most extensively studied immunotherapeutic strategies are immunomodulators, immune checkpoint blockade (ICB), and adoptive cell transfer therapy [14,15,16]. Diverse immunotherapeutic approaches have been employed in clinical trials for various cancers since 2010, yielding promising results [17,18,19,20]. Notably, the immune response triggered by immunotherapy can persist for a long time, even after therapeutic cessation [21]. However, the limited therapeutic efficacy of immunotherapy in solid tumors, including oligodendrogliomas, remains a significant challenge.

**Figure 1:**
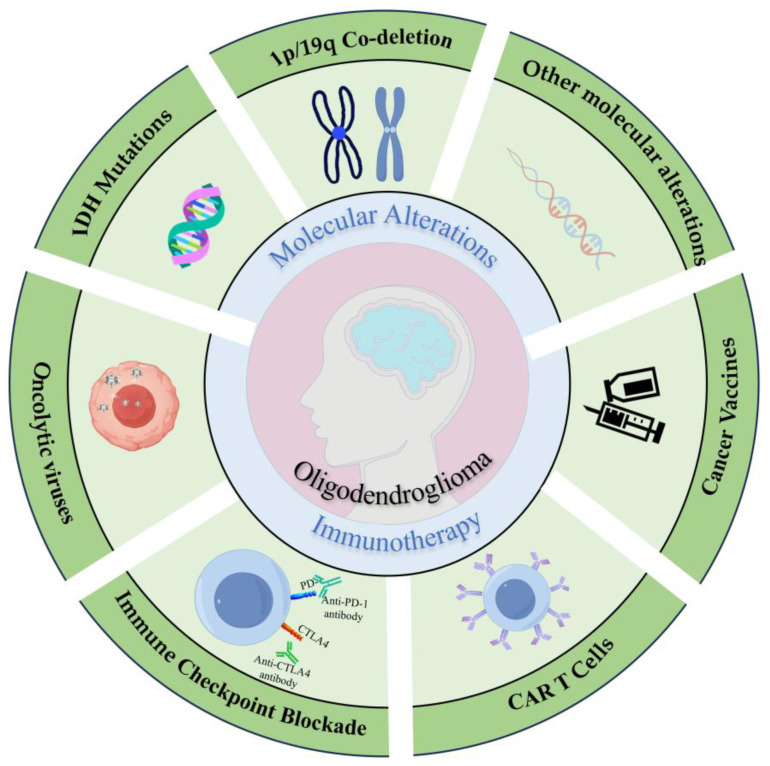
Molecular landscape and immunotherapeutic approaches for oligodendrogliomas. Oligodendrogliomas are molecularly characterized by canonical *IDH1/2* mutations and 1p/19q co-deletion, complemented by secondary alterations in *TERT* promoter and *CIC* genes. Current immunotherapeutic research focuses on four key strategies: oncolytic viruses, immune checkpoint blockade (e.g., PD-1/CTLA-4 inhibitors), CAR T-cell therapy, and tumor-specific vaccines.

Herein, this review aims to enhance diagnostic accuracy and establish a molecular basis for targeted immunotherapies by discussing molecular alterations in oligodendroglioma. Subsequently, we focus on the oligodendroglioma TME, highlighting emerging immunotherapeutic modalities such as oncolytic virus therapy, ICB therapy, chimeric antigen receptor (CAR) T-cell therapy, and cancer vaccines (Figure 1).

## 2. Molecular Alterations

As a distinct type of primary CNS tumors, oligodendrogliomas are initially recognized for their favorable prognosis compared to astrocytic tumors of similar grades based on histopathology [22]. Subsequent studies revealed that oligodendrogliomas exhibit superior sensitivity to chemotherapy with procarbazine, lomustine, and vincristine (PCV chemotherapy) [22]. Clinical investigations have linked their notable response to PVC chemotherapy and prolonged survival to the co-deletion of chromosome arms 1p and 19q in typical anaplastic oligodendrogliomas until the late 20th century, proposing the molecular characterization of oligodendroglioma [23]. With the advancement in large-scale genome sequencing, additional important molecular alterations, such as mutations in the *IDH1/2* genes, were identified in the majority of oligodendrogliomas [23]. The 2021 WHO CNS classification categorizes adult diffuse gliomas into three main groups: (1) oligodendrogliomas (approximately 7% of cases, characterized by *IDH* mutations and 1p/19q co-deletion; (2) *IDH*-mutant astrocytoma (around 11%), defined by *IDH* mutations without 1p/19q co-deletion; and (3) *IDH*-wildtype glioblastomas (approximately 82%) [24].

### 2.1. IDH Mutations

**Figure 2:**
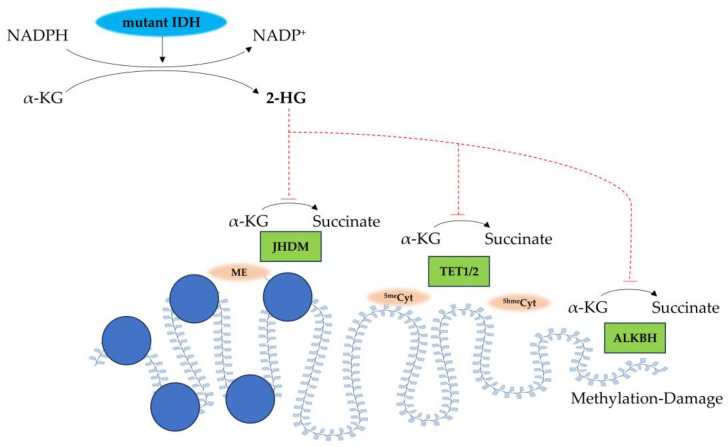
Oncogenetic effects of mutant *IDH1/2*-mediated 2-HG production. Mutant *IDH1/2* catalyzes the NADPH-dependent reduction of α-ketoglutarate (α-KG) to produce the oncometabolite D-2-hydroxyglutarate (2-HG) while oxidizing NADPH to NADP^+^. 2-HG functions as a competitive inhibitor of α-KG-dependent dioxygenases, including: (1) Jumonji C-domain histone demethylases (JHDMs), leading to aberrant histone hypermethylation; (2) ten-eleven translocation (TET) family enzymes (TET1/2), resulting in altered DNA methylation patterns; and (3) ALKBH DNA repair enzymes, impairing the repair of methylation damage. This multifaceted epigenetic dysregulation contributes to the malignant transformation characteristic of *IDH*-mutant gliomas. Me: methylated lysine residue on histone; JDHM: Jumonji C domain-containing histone demethylase; ^5me^Cyt: 5-methylcytosine; TET1/2: ten-eleven translocation enzymes 1/2; ^5hme^Cyt: 5-hydroxymethylcytosine; ALKBH: α-KG-dependent alkB homolog.

*IDH* mutations were first identified in 2008 as a molecular hallmark of diffuse gliomas, and genomic analysis has revealed that nearly 100% of oligodendrogliomas carry *IDH* mutations [23]. *IDH1 R132H* mutation is recognized as the most common mutation, and can be detected using immunohistochemical staining [25]. These mutations occur early in glioma development, and raise the production of oncometabolite D-2-hydroxyglutarate (2-HG), which could be detectable via magnetic resonance spectroscopy (MRS) in vivo [26]. Wild-type *IDH1/2* enzymes catalyze the oxidative decarboxylation of isocitrate to α-ketoglutarate (α-KG), generating agent NADPH as a crucial byproduct for cellular redox homeostasis. In contrast, mutant *IDH1/2* acquires a neomorphic activity that reduces α-KG to the oncometabolite 2-HG while consuming NADPH [27]. Structurally, 2-HG differs from α-KG only through the replacement of a keto group with a hydroxyl group at the C2 position, enabling it to function as a potent competitive inhibitor of α-KG-dependent dioxygenases [27], resulting in widespread DNA hypermethylation and the emergence of a CpG island methylation phenotype (G-CIMP) in gliomas (Figure 2) [26].

This epigenetic reprogramming drives transcriptional alterations, metabolic dysregulation, cellular dysfunction, and impaired differentiation in glia cells [24]. *IDH* mutations are also linked to histone hypermethylation and reduced chromatin accessibility, contributing to differentiation arrest in affected tumor cells, such as oligodendroglioma cells and astrocytoma cells [28]. Moreover, Schvartzman et al. demonstrated that *IDH*-mutant cells exhibit increased heterochromatin, slower DNA replication, and elevated heterochromatin-related replication stress [28]. Both *IDH*-mutant gliomas, oligodendrogliomas and astrocytomas, were reported to commit to the oligodendrocyte lineage, and may be blocked during the premyelination phase due to hypermethylation and downregulated expression of myelination regulators and myelin components [29]. In oligodendrogliomas, *IDH1* gene mutations are strongly associated with dysfunctions of cellular cytosol and peroxisomes, while *IDH2* mutations are only detectable in a small number of oligodendrogliomas that do not carry *IDH1* mutations [30]. *IDH*-mutant gliomas are associated with a more favorable prognosis compared to *IDH*-wildtype tumors [31]. Cairncross et al. once reported that patients with *IDH*-mutant gliomas are more likely to benefit from a combination of radiotherapy and chemotherapy [32]. Although recent studies suggest that genetic mutations may influence prognosis in molecularly defined gliomas, the specific impact of *IDH* mutations on survival in oligodendrogliomas remains understudied [33].

### 2.2. 1p/19q Co-Deletion

This allelic loss, the 1p/19q co-deletion, occurs through a balanced translocation involving the centromeres of chromosomes 1 and 19, resulting in the formation of der(1;19)(q10;p10) and subsequent loss of der(1;19)(p10;q10) [34]. The 1p/19q co-deletion test represents the first molecular diagnostic biomarker in neuropathology since 1994 [35], and this co-deletion is highly specific to oligodendrogliomas [36]. Although its biological implications remain incompletely understood [36], researchers have indicated that recurrent anaplastic oligodendrogliomas with 1p/19q co-deletion may exhibit a better response to PCV chemotherapy [32].

The Far Upstream Binding Protein 1 (*FUBP1*) gene, located on chromosome 1p, is a relatively understudied DNA-binding protein. FUBP1 may function as a DNA-binding and transcriptional regulation protein. For instance, FUBP1 could regulate the transcription of target genes by binding to the Far Upstream Element (FUSE) in the promoter regions of genes, and FUBP1 is also known as a key regulator of the proto-oncogene *MYC*, promoting *MYC* expression through binding to the FUSE region of *MYC* [23,37]. FUBP1 is also involved in RNA metabolic processes, including RNA splicing, stability, and translational regulation [37]. *FUBP1* mutation could be observed in approximately 15~30% of tumors with 1p/19q co-deletion [6]; however, its specific functional role in oligodendroglioma pathogenesis remains unclear [23].

Several methods are available to detect 1p/19q co-deletion, though no single approach is universally accepted as the gold standard [38]. Brandner et al. demonstrated that, compared to the fluorescence in situ hybridization (FISH) and polymerase chain reaction (PCR)-based loss of heterozygosity (LOH) analysis, several acknowledged methods, including chromogenic in situ hybridization (CISH), PCR, real-time PCR, and multiplex ligation-dependent probe amplification (MLPA), exhibit similar high sensitivities except for G-banding [38]. Notably, next-generation sequencing (NGS) and single-nucleotide polymorphism (SNP) arrays show higher specificities compared to FISH and PCR-based LOH assays, suggesting their potential as alternative diagnostic tools [38]. Additionally, MRS could identify elevated levels of cystathionine in vivo, a precursor of cysteine, in 1p/19q co-deleted gliomas compared to non-1p/19q-co-deleted gliomas and normal brain tissues, suggesting another potential biomarker of oligodendrogliomas [39]. Metabolomic analysis of glioma tissue samples has further validated this finding, linking cystathionine accumulation to distinct metabolic flux profiles associated with hemizygous loss of chromosome arm 1p [39].

### 2.3. Other Molecular Alterations

G-CIMP and *MGMT* promoter methylation: The epigenetic landscapes of G-CIMP and the O^6^-methylguanine-DNA-methyltransferase (*MGMT*) promoter methylation are intrinsically linked through *IDH* mutation-driven pathogenesis [40]. In *IDH* mutant gliomas, neomorphic enzyme activity generates the 2-HG, which competitively inhibits α-ketoglutarate-dependent dioxygenases (e.g., TET family enzymes), leading to genome-wide DNA hypermethylation, the hallmark of G-CIMP [41]. Within this context, *MGMT* promoter methylation emerges as a focal epigenetic event, where CIMP silences the DNA repair gene *MGMT*, thereby enhancing tumor sensitivity to alkylating agents like temozolomide [40]. The *MGMT* gene encodes O^6^-alkylguanine-DNA-alkyltransferase (AGT), a crucial DNA repair enzyme that specifically removes mutagenic O6-alkylguanine adducts from DNA, including the cytotoxic O6-methylguanine lesions induced by alkylating agents [6,42,43]. Notably, G-CIMP-high tumors exhibit near-ubiquitous *MGMT* methylation, whereas *MGMT* methylation occurs sporadically in *IDH*-widetype/G-CIMP-negative gliomas [44]. This hierarchical relationship positions G-CIMP as a global epigenetic driver and *MGMT* methylation as a therapeutically consequential downstream effector, collectively contributing to the favorable prognosis of *IDH*-mutant gliomas. Since almost all 1p/19q-co-deleted oligodendrogliomas demonstrate concurrent G-CIMP and *MGMT* promoter methylation, G-CIMP could serve as a molecular classifier for *IDH*-mutant subgroups, and *MGMT* methylation status may provide actionable guidance for chemotherapy selection, exemplifying the translational synergy between these epigenetic biomarkers [6].

*CIC* Gene Mutations: The Capicua Transcriptional Repressor (*CIC*) gene, located on chromosome 19q, acts downstream of the growth factor receptor signaling pathway by repressing target genes through interaction with DNA regulatory elements [23]. Approximately 70% of oligodendrogliomas harbor *CIC* mutations, which may be associated with poor patient outcomes [45].

*TERT* Promoter Mutations: Abnormal reactivation of Telomerase Reverse Transcriptase (*TERT*) is common in solid tumors, particularly those arising from tissues with low self-renewal capacity, such as melanoma, thyroid cancer, and CNS tumors, including gliomas. *TERT* promoter mutations are frequently observed in glioblastomas (70~80%) and oligodendrogliomas (60~70%) [46].

*TRIM67*: Tripartite Motif Containing 67 (*TRIM67*), a RING E3 ubiquitin ligase belonging to the TRIM family, is highly expressed in the cerebellum [11]. Demirdizen et al. reported that TRIM67 may stimulate cell membrane blebbing, enhance cell motility, and attenuate cell adhesion by activating NOGO-A/RhoGT3/ROCK signaling pathways [11]. TRIM67 overexpression, which may act like an oncogene, was reported to correlate with increased tumor growth, augmented tumor volume, elevated vimentin expression at tumor margins, and decreased overall survival in patient-derived oligodendroglioma-orthotopic implanted mice [11].

*PTPRD* and *CNTNAP2*: Protein Tyrosine Phosphatase Receptor D (PTPRD) is a transmembrane phosphatase with an intracellular catalytic domain [47]. Haploinsufficiency of PTPRD drives carcinogenesis in the RCAS PDGFB/Nestin-tvA mouse model of gliomas, and facilitates the development of oligodendrogliomas [48]. Contactin-associated Protein-like 2 (*CNTNAP2*, also known as *CASPR2*) encodes a transmembrane cell adhesion protein that belongs to the neurotoxin family [49]. As a notably large gene located on chromosome arm 7q, *CNTNAP2* has been identified as a tumor suppressor in diffuse gliomas, with specific mutations documented in oligodendrogliomas [47]. Both *PTPRD* and *CNTNAP2* exhibit recurrent mutations in all subtypes of diffuse gliomas, including oligodendrogliomas. Reduced expression of these genes is associated with unfavorable clinical outcomes [47].

*PIK3CA*: *PIK3CA* mutations, which alter the catalytic subunit of phosphatidylinositol 3-kinase (PI3K), are recurrently identified in oligodendrogliomas, with notable enrichment in recurrent tumors [50,51]. These gain-of-function mutations constitutively activate the PI3K/AKT/mTOR signaling axis in anaplastic oligodendrogliomas, exhibiting a predilection for WHO grade 3 histology and correlating with malignant progression [52]. The causal link between PI3K/AKT/mTOR hyperactivation and oligodendroglial tumor malignant transformation could be established, as evidenced by successful xenograft engraftment exclusively in mutant-bearing specimens [52]. These patient-derived models functionally validate the therapeutic vulnerability of this pathway, highlighting PI3K dual inhibitors and mROTC1/2 blockers as priority candidates for preclinical validation in *PIK3CA*-mutant oligodendroglial subsets [52].

*FXYD2*: The sodium/potassium-transporting ATPase subunit gamma (*FXYD2*) gene is located on chromosome 11q23, encoding the γ subunit of the Na-K-ATPase, modulating ion transport homeostasis through allosteric regulation of the enzyme activity [53]. Its expression demonstrates tumor grade- and subtype-dependent patterns in gliomas. Specifically, FXYD2 expression inversely correlates with malignancy grade, and is higher in oligodendrogliomas, *IDH*-mutant, and 1p/19q-codeleted than astrocytomas. Moreover, its mRNA expression could be an independent prognostic marker for gliomas sensitive to temozolomide (TMZ) chemotherapy, but not in patients with low expression [53].

*HOXD12*: *HOXD12*, a member of the homeobox (*HOX*) gene cluster within the 2q31.1 *HOXD* locus, encodes transcription regulators critical for embryonic telencephalon patterning but exhibits transcriptional silencing in normal adult brains through polycomb repressive complex-mediated epigenetic control [54]. Emerging evidence implicates the *HOXD* locus depression in *IDH*-mutant diffuse gliomagenesis, with *HOXD12* transcriptional reactivation specifically marking aggressive clinical trajectories. Nuechterlein N et al. demonstrated that elevated *HOXD12* expression and genomic hypermethylation are associated with older patient age, shorter survival, and older aggressive oligodendroglioma subtypes, which are independently prognostic of *NOTCH1* and *PIK3CA* mutations, loss of 15q, *MYC* activation, and standard histopathological features [54].

Non-coding RNAs: Dysregulated expression of non-coding RNAs (ncRNAs) contributes to oligodendroglioma pathogenesis by modulating gene expression through various mechanisms, such as epigenetic modifications and chromatin remodeling [55]. Specifically, long non-coding RNAs (lncRNAs), enhancer RNAs (eRNAs), non-coding natural antisense transcripts (ncNATs), and circular RNAs (circRNAs) can directly or indirectly influence nitric oxide synthase (NOS) expression and nitric oxide (NO) production, thereby contributing to oncogenesis [56]. Moreover, ncRNAs can impact the DNA binding activity of the CCCTC-binding factor (CTCF, a chromatin organizer), enhance chromatin accessibility at specific loci within the Senescence-associated Secretory Phenotype (SASP) gene, and facilitate the transcription of inflammatory mediators, potentially promoting malignant progression [55,57]. These mechanisms may collaboratively drive the initiation and progression of oligodendrogliomas.

## 3. Immunotherapy

### 3.1. Immune Microenvironment

At the microscopic level, oligodendroglioma cells are characterized by round nuclei and a clear perinuclear halo, surrounded by delicate capillary networks and focal microcalcification [58]. In CNS WHO grade 3 oligodendrogliomas, additional features such as mitotic activity, angiogenesis, and increased necrosis are commonly observed [58]. The immune milieu within tumors, composed of diverse cell populations such as adaptive immune cells, macrophages, dendritic cells (DCs), NK cells, and other innate immune cells, serves as a critical indicator of tumor progression or regression [59]. Microglia within the TME could enhance the expression of GM-CSF and stromal cell-derived factor 1 (SDF-1), promoting glioma cell proliferation and invasion [60]. The overexpression of PD-L1/PD-L2 on surrounding macrophages may inhibit the activation of effector T cells and suppress adaptive immune responses in gliomas [61]. Neurons further contribute to immunosuppression by secreting vascular endothelial growth factor (VEGF) and expressing CD200, which inhibit T-cell activation and immune responses [62]. Neurons also release the growth factor neuroligin-3 (NLGN3), which facilitates glioma cell proliferation through the PI3K-mTOR signaling pathway [62]. The blood–brain barrier (BBB), composed of pericytes, astrocyte foot processes, extracellular matrix, and vascular endothelial cells, protects the brain from harmful substances, and this protective barrier also restricts the entry of administered medications and peripheral immune cells into the CNS, creating a sanctuary for glioma cells to proliferate and infiltrate [63]. Specifically, the BBB’s unique structure restricts the majority of small-molecular drugs and nearly 100% of large biologics from reaching the brain parenchyma [64]. The presence of BBB, microglia cells, and a unique CNS lymphatic system constitutes a distinct microenvironment for gliomas, escaping regular immune surveillance normally observed in peripheral tissues [65]. The dynamic and intricate interplay between tumor cells and their surrounding microenvironment plays a pivotal role in the initiation and progression of solid tumors [59], and this immunosuppressive nature of the glioma microenvironment significantly limits the immunotherapeutic efficacy [66]. To circumvent these limitations, innovative strategies have been developed to bypass or transiently modulate the BBB, primarily through physical barrier disruption techniques (e.g., focused ultrasound) and engineered nanocarrier systems [64]. Among these, nanomedicine approaches leveraging receptor-mediated transcytosis mechanisms show particular promise for CNS tumor targeting. Currently investigated nanosystems can be categorized into lipid-based carriers, polymeric platforms, and solid lipid nanoparticles [64].

Notably, glioma cells could recruit a variety of immune cells to the tumor site through the secretion of cytokines, chemokines, and growth factors [67]. Local CNS inflammation may trigger microglia to recognize specific antigens and present them to activated lymphocytes in the lymphatic vessels lining the dural sinuses as well as meningeal lymphatic vessels, leading to the infiltration of various immune cells across the BBB and subsequent inflammatory and immune responses [65]. Venteicher et al. have identified two distinct inflammatory expression patterns in *IDH*-mutant gliomas [68]. One pattern is associated with microglia, marked by specific markers including CX3CR1, P2RY12, and P2RY13 [68]. The other pattern is linked to macrophages, characterized by the expression of CD163, TGFBI, and F13A1 [68]. Notably, macrophages infiltrating the TME can adopt a microglial-like expression profile under the influence of the phenotypic cues from tumor cells, and the expression of macrophage markers significantly correlates with endothelial cell functions and angiogenesis [68]. Fu et al. demonstrated that mononuclear phagocytes and T cells are the predominant immune cell types within the immune microenvironment of anaplastic oligodendroglioma [69]. Glioma-associated microglia/macrophages (GAMs) exhibited pronounced immunosuppressive properties in these samples [69]. Additionally, the proportion of CD4^+^ and CD8^+^ T cells expressing immune checkpoint markers was significantly higher in anaplastic oligodendroglioma tumor tissues compared to peripheral blood mononuclear cells [69]. These factors collectively establish a unique immune microenvironment, which profoundly impacts the therapeutic responses and prognosis of gliomas [67]. However, the heterogeneity of oligodendroglioma TME remains a pivotal challenge in clinical treatment, underscoring the need for further research to develop targeted therapeutic strategies [70].

### 3.2. Immunotherapeutic Approaches in Oligodendrogliomas

Currently, the standard management of oligodendrogliomas involves maximal safe surgical resection followed by adjuvant radiotherapy [7]. Although radiotherapy is integral to oligodendroglioma treatment, it carries unignorable inherent risks [71]. Specifically, radiotherapy exerts its effects by breaking DNA in tumor cells, leading to their death; meanwhile, it can also harm normal cells, particularly when DNA repair mechanisms are impaired [71]. To improve the overall survival and prognosis of oligodendrogliomas, there is an urgent need for more direct, precise, and less toxic therapeutic strategies [7]. This has driven significant interest in immunotherapy as a promising alternative or adjunct to conventional treatments.

#### 3.2.1. Oncolytic Virotherapy

**Figure 3:**
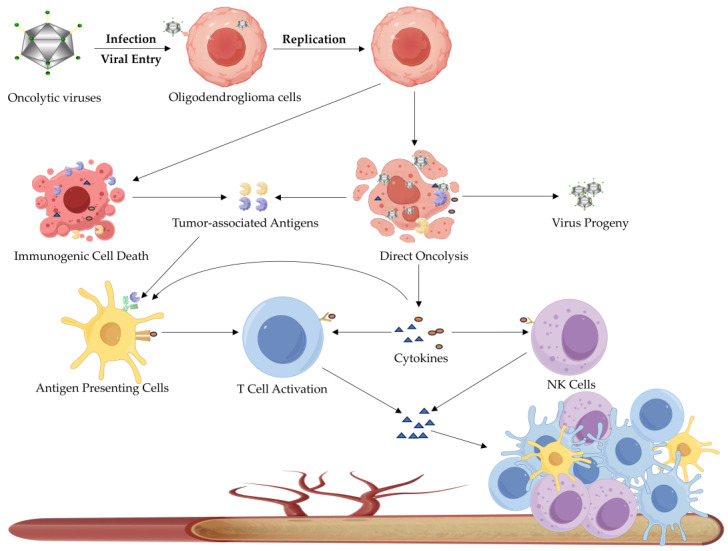
Mechanisms of oncolytic virotherapy (OVT). OVT is an emerging immunotherapeutic approach that utilizes tumor-selective viruses to directly lyse tumor cells while simultaneously activating systemic anti-tumor immunity. The therapeutic process involves: (1) selective viral replication in tumor cells; (2) immunogenic cell death, releasing tumor-associated antigens and viral progeny; (3) initiation of both innate and adaptive immune responses. Viral-mediated tumor lysis generates a pro-inflammatory microenvironment characterized by the release of cytokines and chemokines, thereby recruiting and activating immune effector cells. This dual mechanism of direct oncolysis and immune activation establishes important systemic anti-tumor responses capable of targeting both local and distant tumor metastases.

Oncolytic virotherapy (OVT) is an emerging immunotherapeutic strategy in which oncolytic viruses selectively infect and lyse tumor cells (Figure 3) [72]. Beyond their direct oncolytic effects, these viruses can modulate the TME as well [73]. Specifically, a variety of cellular components, including tumor-associated antigens (TAAs), are released following tumor cell lysis, which may recruit antigen-presenting cells (APCs) to the tumor site [74]. Furthermore, released TAAs may enter the systemic circulation and trigger an anti-tumor immune response in patients [72]. Oncolytic viruses could activate certain signaling pathways, thereby stimulating the secretion of inflammatory cytokines and chemokines and further enhancing the innate immune responses to eliminate infected cells [74]. Moreover, oncolytic viruses, by expressing immunogenic proteins, can convert immunologically “cold tumors” into “hot tumors” [73]. “Cold tumors” are characterized by low immunoreactivity, which limits the immune system’s ability to recognize and eliminate tumor cells effectively [75]. In contrast, “hot tumors” typically exhibit significant immune cell infiltration, facilitating an immune-mediated attack on tumor cells [75].

To enhance their therapeutic potential, oncolytic viruses have been engineered to carry certain cytokines, such as C-C motif chemokine ligand 2 (CCL2), CCL5, CCL19, C-X-C motif chemokine ligand 11 (CXCL11), fibroblast growth factor 2 (FGF2), FMS-like tyrosine kinase 3 ligand (FLT3L), or granulocyte-macrophage colony-stimulating factor (GM-CSF) [76]. These modifications enable oncolytic viruses to selectively target and kill tumor cells while sparing normal tissues, making them a promising treatment for oligodendrogliomas. A phase I clinical trial conducted at University of Alabama at Birmingham (UAB) and Nationwide Children’s Hospital demonstrated that patients with pediatric-type diffuse HGGs showed improved outcomes following intratumoral administration of oncolytic herpes simplex virus type 1 (HSV-1) combined with 5 Gy of radiation; moreover, post-treatment tissue analysis revealed evidence of immune activation, highlighting its therapeutic potential [77]. Chen et al. further elucidated the mechanisms underlying the efficacy of oncolytic viruses in gliomas [78]. They demonstrated that the oncometabolite D-2-hydroxyglutarate (D2HG), produced by mutant *IDH1*, suppresses the expression of Interferon Regulator Factors 3 and 7 (IRF3/7) through a DNA methyltransferase 1 (DNMT1)-dependent mechanism [78]; however, IRF3/7 is a critical transcription factor that may regulate the induction of type I interferon genes and innate immune responses following viral infection [78]. Consequently, the overexpression of mutant *IDH1* or D2HG sensitizes glioma cells to infection by oncolytic viruses such as VSVΔ51, thereby promoting viral replication and enhancing therapeutic efficacy [78]. These findings underscore the significant impact of *IDH1* mutations on antiviral immunity, and suggest that DNMT1 could serve as a predictive biomarker for the OVT responsiveness of *IDH1*-mutant gliomas [78].

#### 3.2.2. Immune Checkpoint Blockade Therapy

Tumor cells employ multiple mechanisms to evade immune surveillance, induce immune tolerance, and resist immune-mediated killing [79]. One such mechanism involves the exploitation of immune checkpoints, which consist of inhibitory and co-stimulatory receptors [80]. For instance, tumor cells often upregulate programmed death-ligand (PD-L1), which binds to the programmed cell death protein (PD-1) on T cells, thereby suppressing T-cell activity [81]. Immune checkpoint inhibitors (ICIs) target these inhibitory receptors on T cells and function by blocking the interaction between checkpoint proteins and their binding partners, preventing the transmission of inhibitory signals, restoring T-cell activation, and enabling T cells to recognize and destroy tumor cells and combat tumors [80,82]. Taken together, ICIs empower the endogenous immune system to target and eradicate tumor cells effectively by alleviating inhibitory signals that impede anti-tumor immunity [81].

Among immune checkpoint molecules, PD-1 and its ligands PD-L1/PD-L2 are the most extensively studied. PD-1 negatively regulates T cell receptor (TCR)-mediated signaling and, upon binding to PD-L1, inhibits cytotoxic T-cell functions and suppresses the production of inflammatory factors, leading to T-cell inactivation [83]. ICIs could disrupt the PD-L1/PD-1 interaction, allowing T cells to regain their cytotoxic potential and eliminate tumor cells [81]. To date, two anti-PD-1 antibodies (nivolumab and pembrolizumab) and three anti-PD-L1 antibodies (atezolizumab, avelumab, and durvalumab) have been approved for clinical use and have demonstrated positive efficacy in treating various solid tumors [83]. However, anti-PD-1 and anti-PD-L1 therapies for gliomas remain in preclinical research and have not yet been approved for clinical application [1]. Another key immune checkpoint molecule is cytotoxic T lymphocyte-associated protein 4 (CTLA-4), which suppresses T-cell co-stimulatory signaling by interacting with CD80 and CD86 ligands on APCs [84]. Preclinical studies have shown that anti-CTLA-4 monotherapy can extend the survival in GL261 syngeneic murine models [85]. Furthermore, recent research has identified several potential biomarkers and combination strategies to enhance the ICB efficacy in gliomas. Zeng et al. discovered that patients with *IDH1*-mutant gliomas may harbor a TME characterized by elevated levels of HLA-DQA2, Homeobox A3 (HOXA3), and Circulating serum amyloid A2 (SAA2), which lead to the suppression of M1 macrophages and CD8^+^ T cells, and are more sensitive to ICB therapy [86]. This suggests that ICB therapy may be particularly effective in *IDH1*-mutant glioma patients with higher expressions of the biomarkers mentioned above [86]. Moreover, Liu et al. reported that the IL-8-CXCR1/CXCR2 signaling could promote angiogenesis and recruit myeloid-derived suppressor cells (MDSCs) within the glioma microenvironment, thereby attenuating the anti-tumor response to ICB therapy [87]. This finding suggests that combining anti-PD-1 therapy with anti-IL-8 agents may enhance the efficacy of ICB therapy [87]. Additionally, Kadiyala et al. demonstrated that combinatorial therapy involving αPD-L1 blockade with an IDH1-R132H inhibitor, ionizing radiation, and temozolomide significantly extended median survival in m*IDH1* glioma-bearing murine models [88]. Notably, this therapeutic strategy not only attenuated T-cell exhaustion, but also promoted the development of memory CD8^+^ T cell populations [88]. Furthermore, Noor et al. demonstrated that increased expression of Podocan-like 1 (PODNL1) and reduced methylation of PODNL1 CpG sites are associated with elevated levels of PD-L1, PD-1, and CTLA4 in LGGs, suggesting that PODNL1 methylation status may serve as a promising biomarker for response to ICB therapy in LGGs [89].

#### 3.2.3. CAR T-Cell Therapy

**Figure 4:**
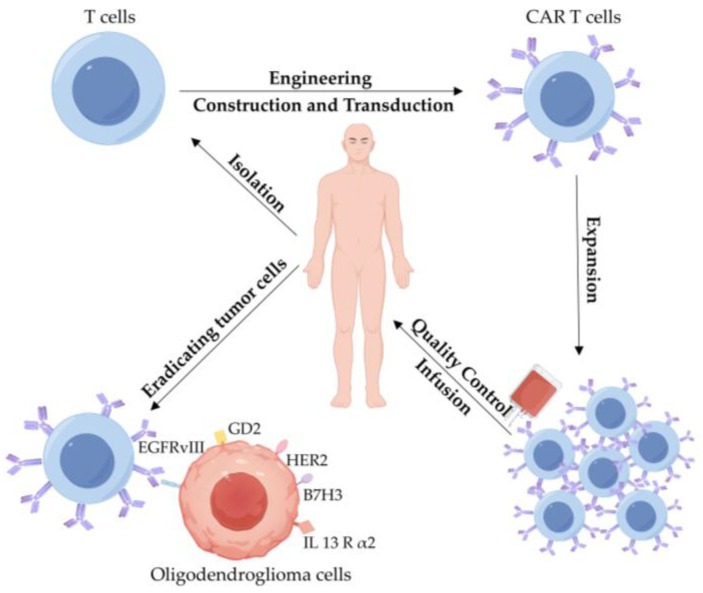
Schematic illustration of chimeric antigen receptor (CAR) T-cell therapy. This innovative immunotherapy involves isolating a patient’s T cells and genetically engineering them ex vivo to express CARs that specifically recognize tumor-associated antigens. These modified CAR T cells can effectively identify and eliminate targeted tumor cells after being reinfused into the patient.

CAR T-cell therapy involves isolating T cells from patients and genetically engineering them in vitro to express chimeric antigen receptors (CARs) on their surface [90]. These CARs are designed to recognize and bind to specific tumor-associated antigens expressed on the surface of tumor cells (Figure 4) [90]. The development of second-generation CARs, which incorporate costimulatory domains such as CD28 or tumor necrosis factor receptor superfamily 9 (4-1BB, also known as CD137), has significantly enhanced the efficacy and persistence of CAR T cells [91]. This advancement, of which six CAR T-cell products target CD19 and B-cell maturation antigen (BCMA) for the treatment of hematological malignancies, has been approved by the Food and Drug Administration (FDA) in USA [91].

Despite the remarkable success of CAR T-cell therapy in treating B-cell leukemia and lymphoma, its application in solid tumors, including CNS tumors, faces several limitations [92]. These challenges include cytokine release syndrome (CRS), limited anti-tumor efficacy, and tumor antigen escape [92]. In particular, CNS tumors present unique challenges due to the distinctive immunological microenvironment and complex interactions with the immune system [93]. Current preclinical research and early-phase clinical trials are primarily focusing on CNS WHO grade 3 or 4 gliomas, with a key emphasis on identifying optimal antigen targets [93]. An ideal target for CAR T-cell therapy should exhibit high and consistent expression in tumor tissues while showing minimal to no expression in healthy tissues to avoid off-target toxicity [93]. Several candidate antigens are under investigation for their potential in glioma therapy: IL13Rα2 is highly expressed in WHO grade 4 gliomas; epidermal growth factor receptor variant III (EGFRvIII) is frequently expressed in gliomas, including glioblastoma (GBM); human epidermal growth factor receptor 2 (HER2) is overexpressed in various CNS tumors; GD2 is highly expressed in pediatric gliomas; and B7H3 is highly expressed in both pediatric and adult gliomas [91]. It is noteworthy that these antigens are either minimally expressed or absent in normal brain tissue, making them promising targets for CAR T-cell therapy in gliomas [91].

#### 3.2.4. Cancer Vaccines

Unlike conventional immunotherapies that primarily target surface antigens, cancer vaccines can target intracellular antigens, broadening their therapeutic potential [94]. Various types of cancer vaccines have been developed, including peptide vaccines, whole tumor cell vaccines, dendritic cell (DC) vaccines, DNA vaccines, and mRNA vaccines [90]. Cancer vaccines aim to elicit systemic immune responses against mutated or highly expressed tumor antigens, such as EGFR, EGFRvIII, NF1, TERT, PDGFRA, PTEN, RB1, IDH1, TP53, PIK3R1, and PIK3CA in gliomas [1]. To date, the FDA has approved sipuleucel-T (PROVENGE), a therapeutic vaccine for prostate tumors, which has been shown to extend patient survival by four months [95].

Peptide vaccines contain specific epitopes, typically consist of 8~25 amino acids, and serve as antigenic targets [96]. They are often conjugated with carrier proteins to enhance immunogenicity, and their simple structure makes manufacturing and storage easier compared with other types of cancer vaccines [96]. Pollack et al. demonstrated that the peptide vaccines of glioma-associated antigens (GAAs) are well-tolerated in children with recurrent LGGs, and GAA vaccine-induced positive immune responses could be observed [94]. Besides, Okada et al. conducted a phase I cohort study to evaluate the safety and immunogenicity of a synthetic peptide vaccine targeting GAA epitopes, including IL-13Rα2, EphA2, WT1, and Survivin, by subcutaneous injection in patients with WHO grade 2 gliomas [97]. They indicated that this treatment was well tolerated, and could induce immune responses against GAAs and improve progression-free survival in these patients [97]. Moreover, Platten et al. carried out a multicenter study that recruited IDH1 (R132H)-positive patients administering an IDH1 (R132H)-specific peptide vaccine [98]. They found that the vaccine-induced immune response could be observed in 93.3% of patients, with 3-year progression-free survival and overall survival rates of 63% and 84%, respectively [98]. Similarly, the multipeptide IMA950 vaccine, consisting of nine HLA-A2-restricted epitopes, two HLA-DR-restricted epitopes, and synthetic epitopes from the hepatitis B virus, is designed to induce robust anti-tumor immune responses in glioma patients [99]. Saijo et al. enrolled 14 patients aged 18 years or older with newly diagnosed or recurrent CNS WHO grade 2 LGGs, who were treated with the IMA950 vaccine in combination with varlilumab [3]. Varlilumab (CDX-1127), a fully human monoclonal agonistic antibody targeting CD27, can mediate CD70-CD27 co-stimulation and activate T cells in the presence of TCR [100]. They reported a significant increase in anti-IMA950 CD8^+^ T-cell response in the peripheral blood from all patients post-vaccination; however, IMA950-reactive CD8^+^ T cells were not detected in resected tumor tissues [3]. Similarly, mass cytometry analyses demonstrated an induced differentiation of Th1 effector memory CD4^+^ and CD8^+^ T cells in peripheral blood mononuclear cells (PBMCs), but not in the TME after the combination therapy of IMA950 and varlilumab [3].

Another kind of neoadjuvant vaccination, GBM6-AD (an allogeneic glioblastoma stem cell lysate) and poly-ICLC (polyinosinic-polycytidylic acid stabilized with poly-lysine and carboxymethylcellulose), was applied in patients with WHO grade 2 gliomas by Ogino et al. in a pilot study to evaluate its safety and immunological effects [101]. The results showed that serum concentrations of CXCL10, IFN-γ, TNF-α, and IL-10 were significantly elevated post-vaccination, accompanied by an increase in activated CD8^+^ T cells in peripheral blood, suggesting that this neoadjuvant vaccination can induce immune responses against GBM6-AD in these WHO grade 2 glioma patients [101]. Moreover, the single-cell RNA/T cell receptor sequencing was introduced to detect the phenotype of CD8^+^ T-cell clones that migrated into the TME in response to this neoadjuvant vaccination, and the amplified effector phenotype of CD8^+^ T cells could be observed [101]. Additionally, mass cytometry analysis further revealed an increase in tissue-resident CD8^+^ T cells with an effector memory phenotype in the TME after vaccination [101]. Furthermore, DC vaccines have been more widely studied in treating other glioma subtypes rather than oligodendrogliomas [1]. DCs play a pivotal role in initiating both innate and adaptive immune responses, making them a promising immunotherapeutic strategy that could also be explored for oligodendroglioma treatment [1].

Currently, most cancer vaccines utilize known antigens, including commonly expressed antigens in the majority of patients, or personalized antigens specific to individual patients [102]. Upon administration, these antigens are presented by APCs to naïve or memory T cells, and then the primed T cells could subsequently migrate to the tumor site, initiating tumor regression and establishing a durable memory response against tumor recurrence [102]. Therefore, peptide-based vaccines have been the most extensively investigated vaccines, and current research on vaccine-based immunotherapy for oligodendrogliomas has predominantly focused on peptide vaccines, partially owing to their well-defined antigenic targets (e.g., IDH1-R132H neoantigens) and manageable safety profiles [1,98]. However, while peptide vaccines demonstrate promising immunogenicity, their clinical efficacy remains limited by several factors, such as intratumoral heterogeneity, weak T-cell infiltration, and immunosuppressive TME [103]. To fully harness the potential of vaccine-mediated immunotherapy, future studies should prioritize exploring alternative vaccine modalities, including DC vaccines and mRNA-based vaccines [60,90]. A multimodal vaccine strategy, potentially integrating these approaches with ICB or targeted therapies, may be pivotal in overcoming the current therapeutic plateau in oligodendrogliomas. Diversifying vaccine platforms will not only address tumor escape mechanisms, but also unlock synergistic anti-tumor immunity, ultimately improving patient outcomes [103].

## 4. Conclusions and Future Directions

Recurrent molecular genetic alterations, such as *IDH1/2*, *FUBP1*, *CIC*, and *TERT* promoter mutations, have been identified to co-occur with 1p/19q co-deletion in oligodendrogliomas [6]. These molecular signatures provide a robust foundation for the consistent diagnosis and classification of glioma subtypes [6]. A comprehensive understanding of these genetic alterations enables precise glioma subclassification, thereby guiding the rationale for subsequent adjuvant treatment. Notably, in patients with grade 2 oligodendroglioma harboring *IDH* mutation, 1p/19q co-deletion, and *CIC* mutation, the administration of PCV chemotherapy following radiotherapy has demonstrated significant survival benefits [7].

Despite its relatively indolent progression, oligodendroglioma is associated with a poor long-term prognosis, with a 20-year overall survival rate of only 37% for grade 3 gliomas [104]. Given that the majority of cases occur in young or middle-aged individuals, there is an urgent need to explore novel therapeutic strategies to improve patient outcomes. Tumor immunotherapy, which harnesses the immune system to target and eliminate tumor cells based on tumor-associated antigens, offers greater precision compared to conventional therapies, thereby minimizing damage to healthy tissue [90]. While immunotherapy has shown remarkable success in the treatment of various hematological and solid malignancies, its application in oligodendroglioma remains largely experimental. Preclinical studies have demonstrated promising results, but the immunosuppressive TME of oligodendroglioma has contributed to suboptimal outcomes in numerous clinical trials, posing significant challenges to clinical efficacy [1]. Therefore, a comprehensive understanding of the oligodendroglioma TME and the development of immunotherapeutic strategies are crucial to address existing limitations.

Future directions in oligodendroglioma immunotherapy will likely focus on targeting immunosuppressive mechanisms within the TME, developing novel immunotherapies directed against tumor-specific mutant molecules with anti-tumor potential, and optimizing combination strategies to enhance therapeutic efficacy. A deeper understanding of the dynamic interactions between the TME and immunotherapeutic agents is crucial for advancing treatment paradigms. Given the complexity of tumor immune evasion mechanisms, rational combination approaches will be essential, including immunotherapy–chemotherapy combinations, immunotherapy-targeted therapy regimens, and combinations of distinct immunotherapies, exemplified by the promising strategy of combining IDH1 neoantigen vaccines with PD-1/PD-L1 checkpoint inhibitors to overcome TME-mediated immunosuppression [105]. Importantly, comprehensive molecular profiling of individual tumors will enable precise patient stratification, paving the way for truly personalized immunotherapeutic approaches that maximize clinical benefit.
